# pH-Responsive Release of Ruthenium Metallotherapeutics from Mesoporous Silica-Based Nanocarriers

**DOI:** 10.3390/pharmaceutics13040460

**Published:** 2021-03-28

**Authors:** Minja Mladenović, Ibrahim Morgan, Nebojša Ilić, Mohamad Saoud, Marija V. Pergal, Goran N. Kaluđerović, Nikola Ž. Knežević

**Affiliations:** 1 BioSense Institute, University of Novi Sad, Dr Zorana Đinđića 1, 21000 Novi Sad, Serbia; minja.mladenovic@biosense.rs; 2 Department of Engineering and Natural Sciences, University of Applied Sciences Merseburg, Eberhard-Leibnitz-Strasse 2, 06217 Merseburg, Germany; nebojsa.ilic@tum.de; 3 Department of Bioorganic Chemistry, Leibniz-Institute of Plant Biochemistry, Weinberg 3, 06120 Halle (Saale), Germany; Ibrahim.Morgan@ipb-halle.de (I.M.); Mohamad.Saoud@ipb-halle.de (M.S.); 4 Department of Civil, Geo and Environmental Engineering, Chair of Urban Water Systems Engineering, Technical University of Munich, Am Coulombwall 3, 85748 Garching, Germany; 5 Institute of Chemistry, Technology and Metallurgy, University of Belgrade, Njegoševa 12, 11000 Belgrade, Serbia; marijav@chem.bg.ac.rs

**Keywords:** pH-responsive drug delivery, mesoporous silica nanoparticles, ruthenium-based anticancer drugs, controlled drug delivery, cancer treatment

## Abstract

Ruthenium complexes are attracting interest in cancer treatment due to their potent cytotoxic activity. However, as their high toxicity may also affect healthy tissues, efficient and selective drug delivery systems to tumour tissues are needed. Our study focuses on the construction of such drug delivery systems for the delivery of cytotoxic Ru(II) complexes upon exposure to a weakly acidic environment of tumours. As nanocarriers, mesoporous silica nanoparticles (MSN) are utilized, whose surface is functionalized with two types of ligands, (2-thienylmethyl)hydrazine hydrochloride (H1) and (5,6-dimethylthieno[2,3-d]pyrimidin-4-yl)hydrazine (H2), which were attached to MSN through a pH-responsive hydrazone linkage. Further coordination to ruthenium(II) center yielded two types of nanomaterials MSN-H1[Ru] and MSN-H2[Ru]. Spectrophotometric measurements of the drug release kinetics at different pH (5.0, 6.0 and 7.4) confirm the enhanced release of Ru(II) complexes at lower pH values, which is further supported by inductively coupled plasma optical emission spectrometry (ICP-OES) measurements. Furthermore, the cytotoxicity effect of the released metallotherapeutics is evaluated in vitro on metastatic B16F1 melanoma cells and enhanced cancer cell-killing efficacy is demonstrated upon exposure of the nanomaterials to weakly acidic conditions. The obtained results showcase the promising capabilities of the designed MSN nanocarriers for the pH-responsive delivery of metallotherapeutics and targeted treatment of cancer.

## 1. Introduction

Cancer treatments typically cause a range of side effects [1,2], induced by poor selectivity for cancerous over healthy cells, and researchers are making a significant effort to develop methodologies for controlled and site-specific drug delivery to cancer [3,4]. Towards this end, the application of nanomaterials is seen as very encouraging [5,6], particularly due to the enhanced permeability and retention (EPR) effect, which enables selective accumulation of nanoparticles at tumour tissues [7]. Moreover, some nanomaterials, such as mesoporous silica nanoparticles (MSN) allow the employment of additional cancer-targeting modalities, through devising nanocarriers with stimuli-responsive drug delivery capabilities [8]. Further beneficial attributes of MSN include their large surface area, uniform mesoporosity, tunable morphology, facile surface functionalization and proven biocompatibility [9,10,11].

Ever since the first demonstration of the capabilities of MSN for drug delivery [12], an abundance of studies followed to optimize the efficiency of MSN for cancer therapy [13], their cellular internalization [14], enhancing targeting ability [15], improving endosomal escape [16], etc. Various external and internal stimuli (pH, temperature, electromagnetic field, light radiation, etc.) have been successfully employed for the delivery of anticancer drugs from MSN [17]. Utilization of the differences in extracellular pH between the tumour and healthy tissues [18,19] is a promising approach for designing systems intended for targeted cancer therapy. Different pH-responsive linkers can be employed for the drug release at the desired time and site [20,21], with hydrazone linkage receiving much attention due to its reversible character at weakly acidic conditions [22,23].

A plethora of studies is being directed towards identifying novel effective metal-based anticancer drugs [24,25,26]. Some of the recent research activities have been dedicated to the development of ruthenium cancer therapeutics [27,28,29]. Ruthenium(II) arene complexes typically designated as [Ru(η^6^-arene)(L)X]^n+^, where, beside arene components, a labile leaving group X (usually chlorido ligand) and L an auxiliary bidentate ligand, have been evaluated against a wide range of cancer cells [30,31,32,33,34]. Ru(II) arenes could also be engaged as radiosensitizers for the treatment of colorectal cancer in radiotherapy [35]. Part of the research community is investigating the potential of nanomaterials for loading and delivering Ru complexes [36], as well as their influence on drug activity [37]. Mesoporous Si-based nanoparticles containing covalently linked Ru(II) complexes have been examined for photodynamic therapy of cancer [38,39], construction of photo-responsive drug delivery systems [40,41] and cancer-targeted therapy based on pore-loaded Ru(II) metallotherapeutics [42,43]. MSN-based nanocarriers for pH-responsive release of metal-based drugs have been also studied. For example, Lv et al. utilized MSN coated with a biotin-chitosan conjugate for pH-sensitive release of a Ru(II) N-heterocyclic carbene complex, which occurs due to a conformational change of chitosan molecule [44]. Encapsulation and pH-responsive release of C,N-cyclometalated organoruthenium complex from amino-functionalized MSN was shown to enhance its effectiveness against glioblastoma multiforme cells [42], while pH-responsive release of Ru(II) polypyridyl complexes from RGD-functionalized MSN have been demonstrated to enhance cancer apoptosis [45]. Aminopropyl-functionalized MSN were also demonstrated to enhance the stability of quercetin and improve its penetration into the skin for topical therapy of JR8 melanoma cells [46].

Herein, a novel nanoplatform for controlled delivery of Ru(II) complexes based on MSN is reported. A commercially available dichloro(p-cymene)ruthenium(II) dimer precursor was coordinated as a monomer to the surface of MSN through two types of ligands (2-thienylmethyl)hydrazine hydrochloride (H1) or (5,6-dimethylthieno[2,3-d]pyrimidin-4-yl)hydrazine (H2), which were functionalized on the surface of MSN through acidification-cleavable hydrazone linkers. Exposure to a weakly acidic environment allowed the release of Ru(II)-complexes containing coordinated H1 or H2 ligands, and enabled their cytotoxic activity, as demonstrated in vitro on B16F1 melanoma cells.

**Figure 1:**
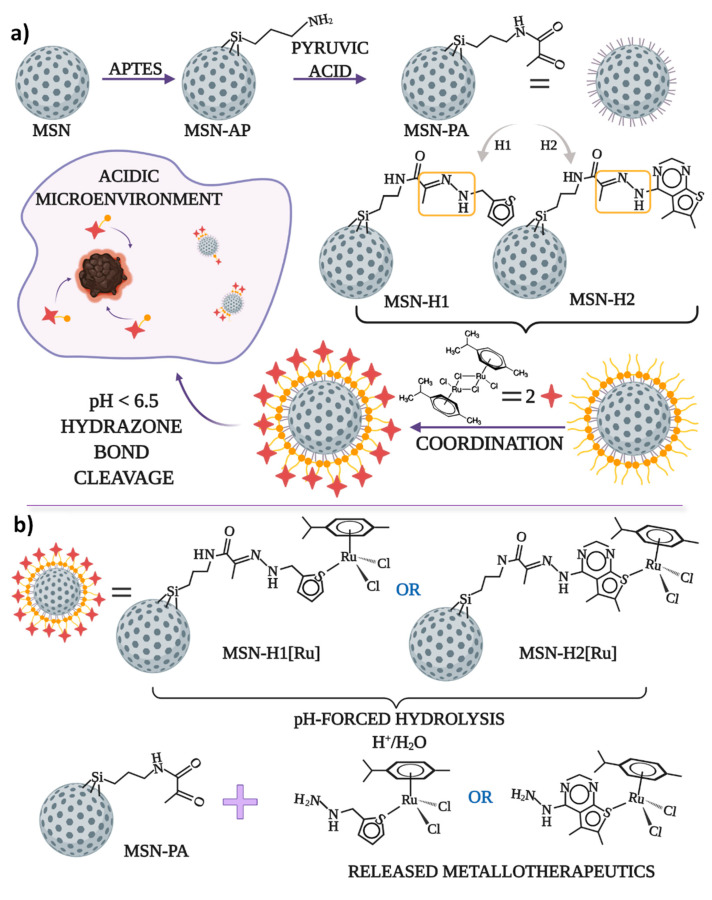
The schematic representation of (**a**) the synthesis process of the anticancer drug delivery systems for pH-responsive delivery of the cytotoxic ruthenium(II) complexes and (**b**) pH-forced hydrolysis of MSN-H1[Ru] and MSN-H2[Ru] and proposed structures of released metallotherapeutics.

Schematic representation of the synthetic steps for functionalization of MSN with pyruvic acid, hydrazone bonding with H1 or H2, coordination of Ru(II) for obtaining final MSN-H1[Ru] and MSN-H2[Ru] materials, as well as the proposed structures of the released Ru(II) complexes upon pH-responsive release, is illustrated in Figure 1.

## 2. Materials and Methods

### 2.1. Materials

N-Cetyltrimethylammonium bromide (CTAB), tetraethyl orthosilicate (TEOS), sodium hydroxide (NaOH), 3-aminopropyltriethoxysilane (APTES), pyruvic acid (PA), N-hydroxysuccinimide (NHS), 1-ethyl-3-(3-(dimethylamino) propyl) carbodiimide hydrochloride (EDC), (2-thienylmethyl)hydrazine hydrochloride, (5,6-dimethylthieno[2,3-d]pyrimidin-4-yl)hydrazine, trifluoroacetic acid (TFA), triethylamine (TEA), ethanol, 2-propanol from Carl Roth (Carl Roth GmbH & Co. KG, Karlsruhe, Germany), dichloro(p-cymene)ruthenium(II) dimer, phosphate buffered saline (PBS), toluene and crystal violet (CV) were purchased from Sigma-Aldrich (Merck KGaA, Darmstadt, Germany). Dulbecco’s Modified Eagle Medium (DMEM) was purchased from Biowest (Riverside, CA, USA). For in vitro experiments nutrition medium RPMI 1640, FCS (fetal calb serum), penicillin/streptomycin and phosphate buffer saline (PBS) were obtained from Capricorn scientific GmbH (Ebsdorfergrund, Germany), glutamine and 3-(4,5-dimethylthiazol-2-yl)-2,5-diphenyltetrazolium bromide (MTT) from GE healthcare (Chicago, IL, USA) and Biomol GmbH (Hamburg, Germany), respectively.

### 2.2. Characterization

The morphology of nanoparticles was characterized by scanning electron microscopy (SEM) on a Tescan Vega3 Phenom (Tescan, Brno, the Czech Republic). The surface areas and pore size distributionswere calculated using the Brunauer-Emmett-Teller (BET) and Barrett-Joyner-Halenda (BJH) method [47], respectively, performed on an Autosorb iQ/ASiQwin (Quantachrome Instruments, Anton Paar, QuantaTec Inc., Boynton Beach, FL, USA). Thermogravimetric analyses (TGA) were performed on a Netzsch TG 209 F1 Iris instrument (Netzsch Holding, Selb, Germany). FTIR (Fourier transform infrared) spectra have been obtained by the Attenuated Total Reflection (ATR) technique using IR spectrometer Bruker Vertex 70 (Bruker, Billerica, MA, USA). Zeta potential measurements were conducted on Zetasizer Ultra (Malvern Panalytical, Kassel, Germany). Dynamic light scattering (DLS) measurements were conducted on a Particle size analyzer—Litesizer 500 (Anton Paar, Graz, Austria). Energy-dispersive X-ray Spectroscopy (EDS) experiments were conducted on Tabletop Scanning Electron Microscope Hitachi TM3030. Small-angle X-ray scattering (SAXS) measurements were performed on a D8 ADVANCE (Bruker) X-ray diffraction system. Inductively coupled plasma optical emission spectrometry (ICP-OES) measurements were performed using a Thermo Scientific iCAP 6500 Duo spectrometer using the following conditions: flush pump rate: 100 rpm, analysis pump rate: 50 rpm, pump stabilization time: 5 s, RF power: 1150 W, auxiliary gas flow: 0.5 L/min, nebulizer gas flow: 0.70 L/min, coolant gas flow: 12 L/min.

### 2.3. Synthesis of MSN and Functionalization with Ligands

MSN were obtained by the sol-gel templating method. In brief, CTAB (1 g) was dissolved in deionized water (480 mL) with the addition of NaOH (2 M, 3.5 mL) at 80 °C. Then, TEOS (5 mL) was added dropwise under vigorous stirring. The reaction was continued at 80 °C for 2 h at 500 rpm. The material was collected by filtration and washed twice with water and once with ethanol. After drying at 80 °C, it was calcinated at 500 °C (ramp 2 deg/min) to remove the surfactant.

The amino-functionalized MSN was prepared through the grafting procedure. MSN (650 mg) was refluxed at 110 °C in anhydrous toluene containing APTES (0.65 mmol) overnight. The material (MSN–AP) was collected by filtration, washed twice with 2-propanol, and dried at 80 °C.

The functionalization with PA was performed in an aqueous solution at pH 6.0. First, the carboxyl groups of PA (960 mg) were activated through stirring for 2 h at room temperature in a solution containing NHS (240 mg) and EDC (480 mg). Then, an aqueous dispersion (pH 6.0) containing MSN-AP (640 mg) was added to the above solution under stirring. The reaction was continued for 24 h at RT. The material (MSN–PA) was collected by filtration, washed with water and ethanol, and dried at 80 °C.

In order to obtain a pH-responsive hydrazone bond, 250 mg of MSN-PA was dispersed in 100 mL of ethanol containing 0.02 M of appropriate hydrazine (H1/H2), followed by the addition of 100 μL of TFA and 2 mL of TEA under stirring. The reaction solution was degassed with nitrogen for 30 min. Afterward, the reaction mixture was protected from light and refluxed at 80 °C for 24 h. The final products (MSN–H1 and MSN–H2) were obtained by filtration, washed with boiled ethanol, and dried at 80 °C.

### 2.4. Reaction MSN Materials with Dichloro(p-Cymene)ruthenium(II) Dimer

Pristine MSN (200 mg), MSN-H1 (200 mg) or MSN-H2 (200 mg) were separately dried under vacuum for 4 h at 100 °C. After cooling to the room temperature, anhydrous toluene (30 mL) and ruthenium metal complex (dichloro(p-cymene)ruthenium(II) dimer, 0.09 mmol) was added under an inert atmosphere and further stirred at 60 °C overnight. Afterwards, materials (MSN[Ru], MSN-H1[Ru] and MSN-H2[Ru]) were collected by filtration and washed 3 times with toluene and dried under vacuum.

### 2.5. Drug Release Investigation

PBS solution (4 mL) with different pH values (7.4, 6.0 and 5.0) containing 4 mg of nanoparticles (MSN[Ru], MSN-H1[Ru] and MSN-H2[Ru]) was stirred at room temperature for 48 h. In order to investigate the drug release kinetics, suspensions were centrifuged at 12,066× *g* rcf at certain time intervals (15 min, 30 min, 1 h, 2 h, 4 h, 24 h, and 48 h) to separate a supernatant (4 mL), which was analysed using UV/VIS spectrophotometer DSH-L6/L6S (Dshing Instrument Co., Zhuhai, China) by measuring absorbance in the range from 250 to 600 nm. After the measurement, materials were re-dispersed in the supernatant and returned to bulk suspension under stirring.

### 2.6. Determination of Cell Viability

B16F1 cells were maintained routinely using a complete medium (RPMI 1640, 10% FCS, 1% glutamine and 1% penicillin/streptomycin) with an atmosphere of 5% CO_2_ at 37 °C. For cell viability assay, the cell suspension was prepared at a density of 5000 cells/100 µL. The stock solutions of the tested materials were prepared at a concentration of 20 mg/mL in PBS (pH 7.2) or acetate buffer (pH 5.0). The stock solutions of prepared materials were incubated on a shaker for 4 h. The materials were tested at different concentrations, prepared by dilution of stock solutions in a completed medium, against mouse melanoma B16F1 cell line and incubated for 48 h at 37 °C and 5% CO_2_. Subsequently, MTT and CV assays were performed according to the literature [48]. The absorbance was measured using plate reader Spectramax (Molecular Devices, San Jose, CA, USA) at 570 and 670 nm. The cell viability is represented as a percentage compared to untreated cells and the mean calculated using a four-parametric logistic function.

## 3. Results and Discussion

**Figure 2:**
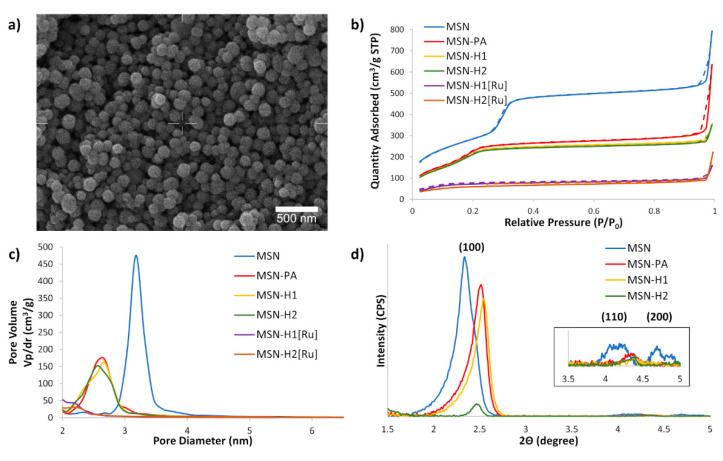
(**a**) SEM micrograph of MSN; (**b**) BET N_2_ adsorption/desorption isotherms of the synthesized materials, with solid and dashed lines referring to the adsorption and desorption processes, respectively; (**c**) BJH average pore diameter distribution for synthesized materials and (**d**) SAXS patterns of prepared nanoparticles. Inset shows higher magnification of (110) and (200) reflections of MSN.

The SEM of MSN (Figure 2a) demonstrated that the synthesized material consists of uniform spherical nanoparticles with diameters in the range of 160 to 220 nm. Nitrogen sorption analysis (Figure 2b) revealed that the initial MSN material possesses a high BET surface area (1046 m^2^/g). The BET isotherms of non-functionalized MSN, as well as for MSN-PA, MSN-H1 and MSN-H2 displayed a typical type IV isotherm without an apparent hysteresis loop, confirming a narrow, uniform, and well-defined mesoporous structure. Though, with the introduction of functional groups and, particularly upon coordination of Ru(II) metal complex, the surface areas, total volumes of mesopores, average and BJH pore diameters decreased (Table 1), evidencing successful functionalization. After functionalization of MSN with PA, the surface area decreased without the influence on the total volume of mesopores, due to the small size of the PA molecule. Upon subsequent functionalization with larger H1 and H2 molecules, the surface area but also the mesopore pore volumes decreased. Further modification of the functional groups by coordination of the Ru(II) complex, leads to the change of the BET isotherm to type II, also followed by a decrease in the BJH pore diameter below 2 nm.

These results indicate blocking of the MSN mesopores after coordination of the Ru(II) complex, which is in agreement with our previous observation that Ru(II) complexes are indeed capable of capping the MSN pores, which was also utilized for entrapping the cargo molecules and their subsequent release by exposure to visible light [40,49]. Small angle X-ray scattering measurements evidence the presence of hexagonally ordered porosity, typical for MCM-41-based MSN, with the following peak positions: (100) at 2.35 degrees (2θ), (110) at 4.17 degrees (2θ) and (200) at 4.70 degrees (2θ) (Figure 2d). Covalent surface functionalization lead to shifting of the peaks to higher Bragg angles due to decreasing pore size and decreased intensity of higher reflections ((110) and (200)) due to disruption of symmetry upon covalent surface modifications.

**Figure 3:**
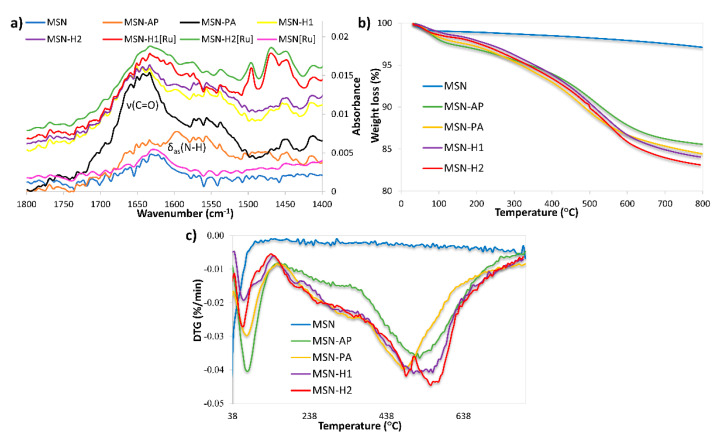
(**a**) FTIR spectra and (**b**,**c**) thermogravimetric analysis of the prepared materials.

FTIR spectroscopy (Figure 3a and Figure S1), thermogravimetric analysis (Figure 3b,c) and zeta potential measurements were further employed to evidence the successful surface functionalization. All FTIR spectra are dominated by bands at 441 cm^−1^ (Si-O rocking vibration), 809 cm^−1^ (internal Si-O-Si symmetric stretching vibration) and 1062 cm^−1^ (internal Si-O-Si asymmetric stretching vibration) [50,51]. In the region 1300 to 1800 cm^−1^ non-functionalized MSN and MSN[Ru] material, which contains the surface adsorbed Ru(II) complex, exhibit similar spectra, with the dominant band at 1640 cm^−1^, characteristic for stretching vibration of surface adsorbed water. This result hints that a small amount of Ru(II) precursor was adsorbed on the MSN surface in the absence of any functionalization and, therefore, no significant changes in the spectra are observed. After grafting with APTES a new band appeared at 1595 cm^−1^ (Figure 3a), assigned to N-H asymmetric bending vibration. The appearance of a new intense band in the region 1600–1700 cm^−1^ in the spectrum of MSN-PA can be ascribed to C=O stretching vibration and evidenced successful functionalization with pyruvic acid. Further functionalization of nanomaterials with H1 and H2 leads to the decrease in the carbonyl group vibration band intensity, suggesting the successful formation of hydrazone linkage with H1 and H2 through the carbonyl group. The spectra of MSN-H1 and MSN-H2 exert similar bands as both materials are dominated by the vibrations of hydrazone linkage and aromatic rings. Successful coordination of the Ru(II) complex was confirmed by the appearance of new bands in regions 690–900 cm^−1^ and 1400–1600 cm^−1^, in the FTIR spectra of MSN-H1[Ru] and MSN-H2[Ru].

The introduction of functional groups onto the surface of MSN was also revealed by thermogravimetric analysis (Figure 3b,c). All the materials show weight loss below 150 °C due to surface-adsorbed water. The weight loss patterns between 150 °C and 750 °C show different bands for different materials, indicating the presence of different surface moieties and successful surface functionalization. Moreover, the amounts of functional group grafted with respect to the starting MSN were 9.87 wt %, 11.22 wt %, 12.45 wt %, and 13.14 wt % in the case of MSN-AP, MSN-PA, MSN-H1, and MSN-H2, respectively. A noticeable agreement in the rise of weight loss with every synthesis step is evidenced.

Changes in zeta potential values further supported the evidence of successful functionalization of MSN. Pristine MSN possesses a negatively charged surface (ζ = −20.6 mV) due to deprotonated silanol groups at neutral pH. The change of zeta potential to positive values after the conjugation of hydrazines (ζ = +47.1 mV for MSN-H1 and ζ = +52.5 mV for MSN-H2) and [Ru] complex (ζ = +64 mV for MSN-H1[Ru] and ζ = +57.1 mV for MSN-H2[Ru]) suggested that these processes were successfully accomplished. DLS measurements revealed that the hydrodynamic diameters of the functionalized nanoparticles, dispersed in water, are centered at 216.2 ± 9.97 nm, 289.9 ± 13.78 nm, and 306.9 ± 0 nm for MSN[Ru], MSN-H1[Ru] and MSN-H2[Ru], respectively (Figure S2). We further determined the hydrodynamic diameters of nanoparticles in culture media (DMEM, 10% FSB), which revealed no substantial difference in the case of the predominant hydrodynamic diameters of MSN-H1[Ru] and MSN-H2[Ru], though the size distribution of nanoparticles was substantially wider. However, the suspension of MSN[Ru] showed different behaviors, i.e., substantial agglomeration of these nanoparticles was evident, with the predominant peak in cell medium shifted to 520 ± 29.76 nm. The results point to the significance of the covalent attachment of metallotherapeutics to the surface of MSN, which enhances the stability of nanoparticles in the physiological environment.

EDS measurements confirmed that MSN[Ru], MSN-H1[Ru], and MSN-H2[Ru] materials contain ruthenium in the amounts of 0.32 wt %, 8.36 wt %, and 7.92 wt %, respectively. Chlorine was also quantified in the atomic ratio Ru:Cl ca. 1:2 (Table S1), which is in agreement with the suggested structure of the coordinated Ru(II) complex. EDS chromatograms are provided in Supplementary Materials (Figure S3).

**Figure 4:**
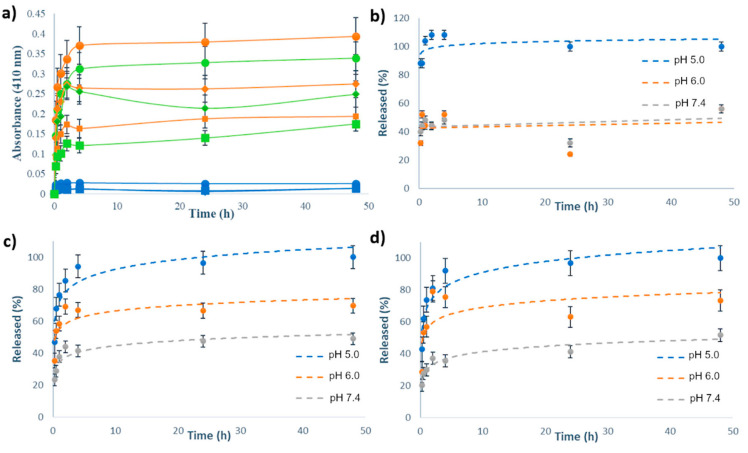
(**a**) Release profiles of Ru(II)-based complexes from MSN-H1[Ru] (orange curves), MSN-H2[Ru] (green curves) and MSN[Ru] (blue curves) in PBS buffers with pH 5.0 (●), pH 6.0 (◆) and pH 7.4 (■) for 48 h, as measured by UV/VIS spectrophotometry at 410 nm; Release kinetics of (**b**) MSN[Ru]; (**c**) MSN-H1[Ru] and (**d**) MSN-H2[Ru] in PBS buffers with various pH values for 48 h, as measured by UV/VIS spectrophotometry at 410 nm, with absorbance values normalized to the absorbance of the supernatant from the same type of the material at 48 h and pH 5.0.

The release kinetics of Ru(II)-complexes were measured from the suspension of MSN[Ru], MSN-H1[Ru] and MSN-H2[Ru] in PBS buffers at different pH values (5.0, 6.0 and 7.4) at room temperature. UV/VIS spectrophotometry was employed to monitor the release process of the metal complexes to the bulk solution, upon separation of MSN by centrifugation, with measuring the absorbance of supernatants at 410 nm. The release kinetics curves are shown in Figure 4, with Figure 4a showing the as-measured absorbances from the supernatants, and Figure 4b–d showcasing the kinetic profiles with absorbance values normalized to the absorbance of the supernatant from the same type of the material at 48 h and pH 5.0.

As can be noted, the release kinetics were clearly pH-dependent with enhanced cargo release upon acidification of the environment. Figure 4a reveals that the amount of released Ru(II) complex in the case of MSN[Ru] was an order of magnitude lower than in the case of MSN-H1[Ru] and MSN-H2[Ru]. This result evidences the crucial role of surface-bound ligands for constructing efficient delivery systems and for achieving the pH-responsive release of these types of Ru(II) complexes. Furthermore, by comparing the release kinetics, it is evident that the drug release from MSN[Ru] reaches its maximum within one hour of measurements, while for the other two materials the release kinetics is evidently slower, reaching the plateau only after 4 h. As the release of Ru(II) complex in the case of MSN[Ru] occurs rapidly due to simple desorption of the adsorbed species, the slower release kinetics in the case of MSN-H1[Ru] and MSN-H2[Ru] supports the assumption that these release processes are governed by a more complex mechanism than desorption, such as the process of hydrolysis of the hydrazone linkages. The measurements at pH 6.0 reveal a stronger initial burst of the cargo release after 2 h of measurements, followed by the decrease in the measured absorbances. This result is not observed at pH 5.0 and may be related to the reversibility of the hydrazone formation [52], which is less favored at lower pH.

UV/VIS spectra of Ru(II) complexes in supernatants after the release kinetics measurements (Figure S4) exhibited different bands, which were highly dependent on the pH values. This result hints at possible hydrolysis and substitution of ligands, giving rise to different possible mononuclear and binuclear Ru(II) complexes coexisting in solution, containing different combinations of Cl, OH and H_2_O ligands [53]. Hence, as the UV/VIS spectra change with pH, comparison of absorbances at the same wavelength may not give a reliable estimation of the concentration of the released Ru(II) complexes due to the probable differences in extinction coefficients at 410 nm. However, the final released amounts of the ruthenium were quantified from the solution by ICP-OES, after 48 h of stirring in solutions of different pH values (Table 2), and the results of this analysis also evidence the beneficial effects of acidification on the release of Ru(II) complexes.

The quantified amount of ruthenium after 48 h of stirring revealed similar amounts of the released Ru for MSN-H1[Ru] and MSN-H2[Ru] at both pH 5.0 and pH 6.0, though these concentrations are significantly higher than the released Ru amounts at pH 7.4. In the case of MSN-H1[Ru], the concentration of Ru was higher by 35% and for MSN-H2[Ru] by 19.5% than the amounts released at pH 7.4. This result can be indeed correlated to the well-documented acid-catalysed hydrolysis of hydrazone moieties, which occurs even at weakly acidic conditions (pH < 6.5) [32,33,34]. When considering the amounts of Ru on the materials, as determined by EDS, the release capacity of the materials at pH 5.0 is 46.9%, 31.4%, and 25.1% of the loaded amount, for MSN[Ru], MSN-H1[Ru] and MSN-H2[Ru], respectively. Hence, even though similar amounts of Ru(II) complex were coordinated to MSN-H1 and MSN-H2, the lower amount of H2[Ru] was released at all investigated pH, in comparison to the release of H1[Ru]. This result might be ascribed to lower solubility of H2[Ru] in an aqueous environment as H2 ligand contains an additional pyrimidine ring in comparison to H1.

**Figure 5:**
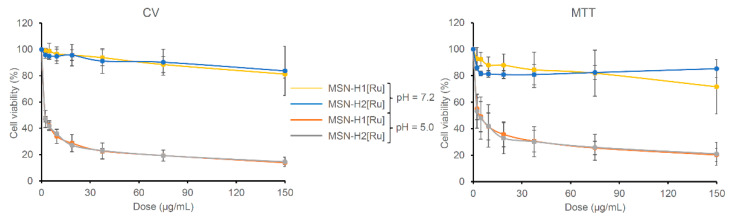
Viability of the B16F1 cells determined with CV and MTT assays treated (48 h) with different concentrations of investigated MSN on pH 5.0 and 7.2.

To evaluate the potential of prepared nanomaterials for cancer treatment, in vitro cell viability experiments were performed against B16F1 melanoma cell lines. The materials were first incubated for 4 h in buffers at pH 5.0 and 7.2 and then different dilutions of the materials in the medium were prepared for treating the cells for 48 h. The half maximal inhibitory mass concentration (MC_50_) values of Ru(II)-functionalized MSN, calculated as a mass concentration (µg/mL) of the Ru(II)-containing MSN needed to inhibit the cell viability by 50%, are listed in Table 3, while dose-dependent results of B16F1 cells treated with [Ru] immobilized on MSN are shown in Figure 5.

As can be seen, the investigated materials showed high activity upon pre-incubation of materials in an acidic environment (pH 5.0), while after pre-incubation at pH 7.2 materials were found inactive against B16F1 cells. This substantial difference can be evidently associated with the cleavage of hydrazone bonds and drug release differences from the tested materials. Results from the MTT and CV assays indicate that both nanomaterials are slowing the metabolic profile of the cells. Comparing to the MC_50_ values of mesoporous silica loaded with cisplatin (CV assay: MC_50_ = 1.23 ± 0.13 μg/mL), materials reported herein, preincubated for 4 h at pH 5.0, showed two times higher potential against B16F1 cells [54]. The MC_50_ values of MSN-H1[Ru] and MSN-H2[Ru] against the B16F1 cells are not statistically different. Though, as MSN-H1[Ru] releases more Ru(II) species than MSN-H2[Ru], the viability measurements give an indication of a possibly higher potency of H2[Ru] in comparison to H1[Ru] against this type of cells. In comparison, the control experiment with pristine MSN showed that it does not affect cellular viability (MC > 100 µg/mL) at both pH values (Figure S5). Furthermore, previous research showed that starting Ru(II) precursor for the preparation of MSN-metallotherapeutics did not exhibit cytotoxicity against different cell lines, such as human colon adenocarcinoma (Colo205 and its multidrug-resistant counterpart Colo320), as well as human embryonal lung fibroblast cell line (MRC-5) [55]. Such results strengthened our conviction that functionalization of MSN with coordination-capable ligands, such as H1 and H2, improves the loading capacity, but also enhances the cytotoxic activity of Ru(II) metallotherapeutic though pH-responsive release of H1- and H2-containing Ru(II) complexes.

## 4. Conclusions

In summary, we constructed two types of mesoporous silica nanoparticle-based nanocarriers, containing surface-attached ligands and coordinated Ru(II)-based metallotherapeutic. The ligands were attached to MSN through a pH-responsive hydrazone linkage and the enhanced release of Ru(II) complexes was successfully achieved at weakly acidic conditions in comparison to the release at physiological pH. In Vitro evaluation of the prepared materials against B16F1 cells evidenced their potent anticancer activity upon exposure to weakly acidic conditions, which is encouraging toward further investigation in utilization of functionalized MSN as novel cancer-targeting nanotherapeutics for pH-responsive delivery of cytotoxic Ru(II) complexes.

## Figures and Tables

**Table 1:** BET surface area, mesopore volume values, average and BJH pore diameter.

Material	BET Surface Area (m^2^/g)	Total Volume of Mesopores (cm^3^/g)	Average Pore Diameter (nm)	BJH Pore Diameter (nm)
MSN	1046	0.6	3.4	2.7
MSN-PA	753	0.6	2.8	2.2
MSN-H1	703	0.2	2.5	2.2
MSN-H2	696	0.2	2.5	2.1
MSN-H1[Ru]	298	0.1	2.2	<2
MSN-H2[Ru]	238	0.2	2.5	<2

**Table 2:** The amounts of ruthenium (µg/mg MSN) released after 48 h of stirring, as measured by ICP-OES.

Material	pH 5.0	pH 6.0	pH 7.4
MSN[Ru]	1.50 ± 0.01	0.95 ± 0.02	0.64 ± 0.01
MSN-H1[Ru]	26.28 ± 0.13	26.13 ± 0.08	19.47 ± 0.17
MSN-H2[Ru]	19.91 ± 0.11	19.60 ± 0.06	16.66 ± 0.03

**Table 3:** MC_50_^1^ values [µg/mL] of the B16F1 cells treated for 48 h with MSN-H1[Ru] or MSN-H1[Ru].

Material	pH 7.2		pH 5.0	
	MTT Assay	CV Assay	MTT Assay	CV Assay
MSN-H1[Ru]	>100	>100	4.00 ± 0.86	0.63 ± 0.15
MSN-H2[Ru]	>100	>100	2.49 ± 1.02	0.63 ± 0.17

^1^ MC_50_—The half maximal inhibitory mass concentration of the material.

## Data Availability

The data presented in this study are available in the research article and supplementary material here.

## Supplementary Materials

The following are available online at https://www.mdpi.com/article/10.3390/pharmaceutics13040460/s1, Figure S1: Full range FTIR spectra of the synthesized materials, Figure S2: Particle size distribution of Ru-modified nanoparticles in water (top) and culture medium (bottom), Table S1: Variation of ruthenium and chloride concentrations from EDS measurement, Figure S3. EDS chromatograms of (a) MSN-H1[Ru]; (b) MSN-H2[Ru] and (c) MSN[Ru] with insets representing Ru mapping, Figure S4: UV/VIS spectra of supernatants at different pH values after 48 h of stirring, Figure S5: Viability of the B16F1 cells determined with CV and MTT assays treated (48 h) with pristine MSN on pH 5.0 and 7.2.
