# Supplementary material for: pH-Responsive Release of Ruthenium Metallotherapeutics from Mesoporous Silica-Based Nanocarriers
Source: Pharmaceutics. 2021 Mar 28;13(4):460. doi: 10.3390/pharmaceutics13040460 (PMC8067187; doi:10.3390/pharmaceutics13040460)
Supplement: Supplementary file 1 [file pharmaceutics-13-00460-s001.pdf]

pharmaceutics

MDPI

Supplementary Materials: pH-Responsive Release of Ruthenium Metallotherapeutics from Mesoporous Silica-Based Nanocarriers

Minja Mladenović, Ibrahim Morgan, Nebojša Ilić, Mohamad Saoud, Marija V. Pergal, Goran N. Kaluđerović and Nikola Ž. Knežević

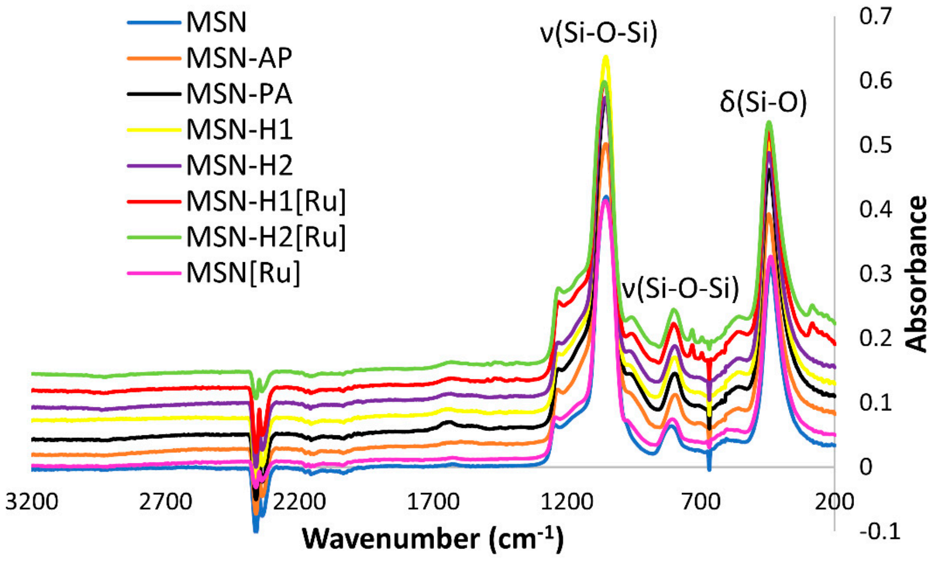

Figure S1. Full range FTIR spectra of the synthesized materials.

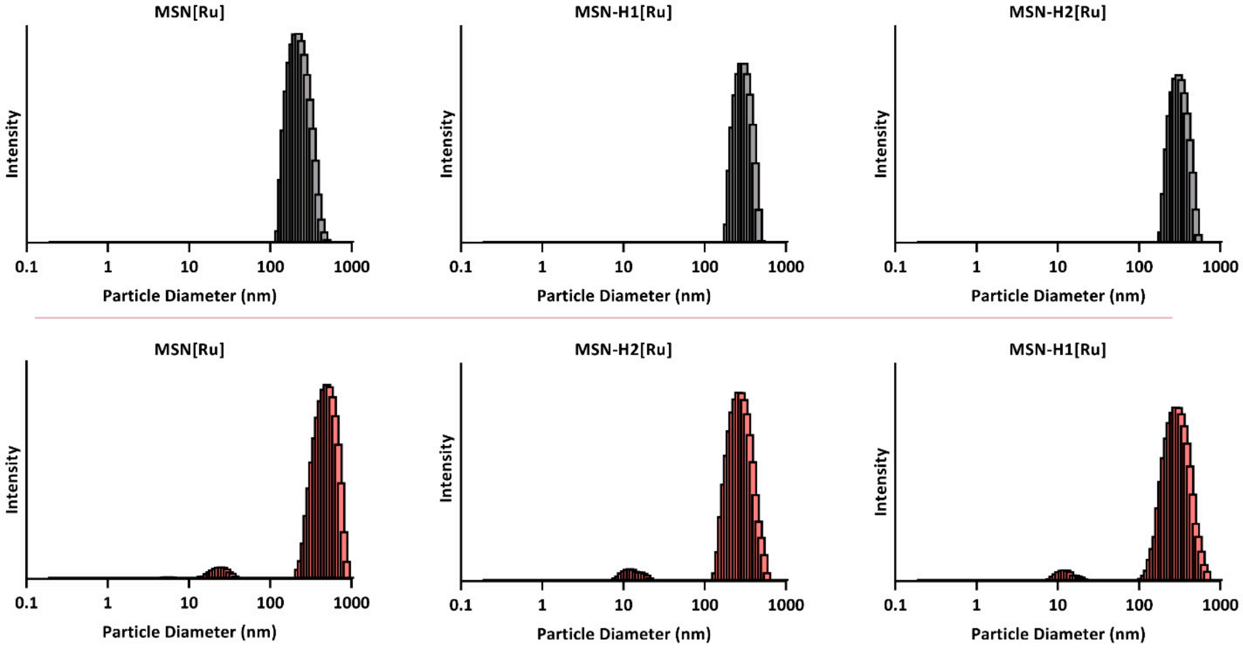

Figure S2. Particle size distribution of Ru-modified nanoparticles in water (top) and culture medium (bottom).

Table S1. Variation of ruthenium and chloride concentrations from EDS measurement.

Material	Ruthenium		Chloride	
	norm. C [wt.%]	Atom. C [at.%]	norm. C [wt.%]	Atom. C [at.%]
MSN[Ru]	0.315 ± 0.25	0.08 ± 0.075	0.314 ± 0.10	0.187 ± 0.069
MSN-H1[Ru]	8.361 ± 0.92	1.884 ± 0.25	7.287 ± 0.51	4.68 ± 0.45
MSN-H2[Ru]	7.916 ± 0.92	1.706 ± 0.26	6.239 ± 0.59	3.85 ± 0.49

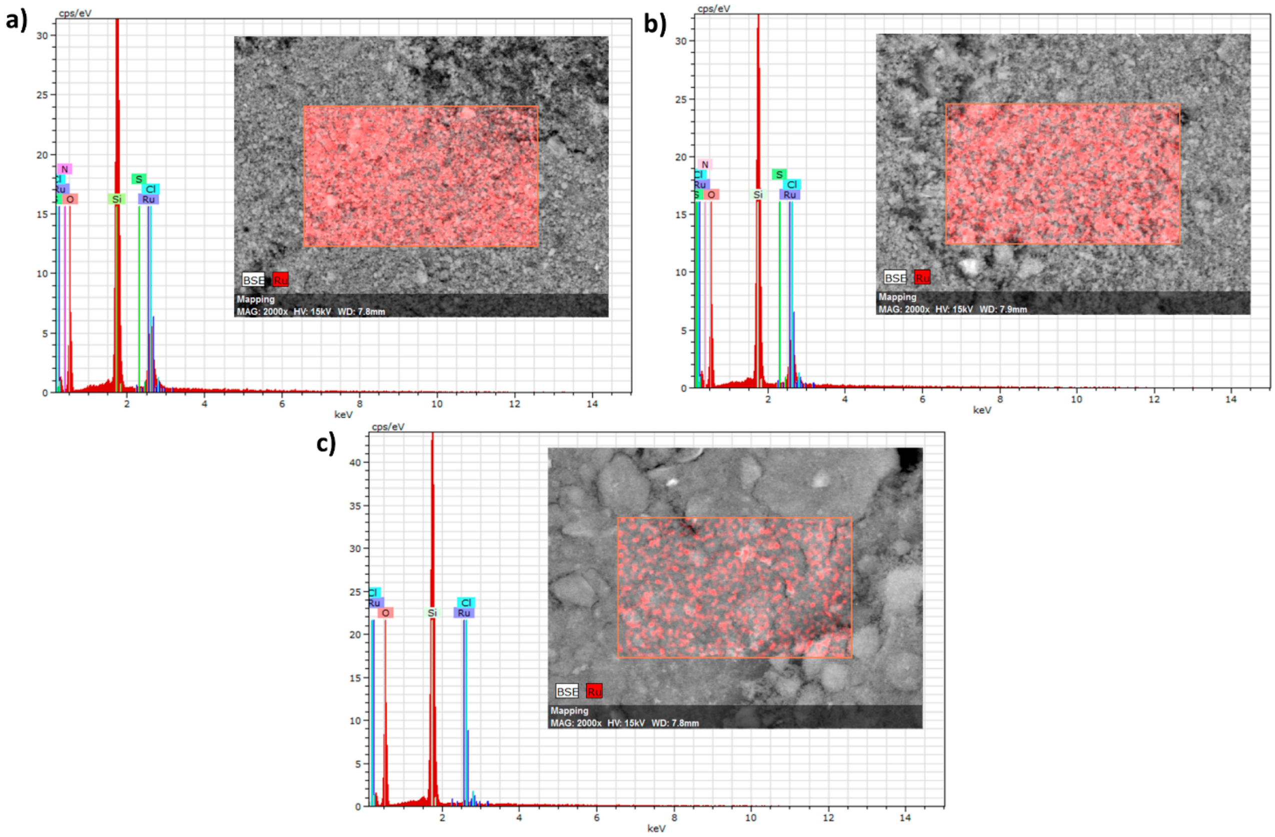

Figure S3. EDS chromatograms of (a) MSN-H1[Ru]; (b) MSN-H2[Ru] and (c) MSN[Ru] with insets representing Ru mapping.

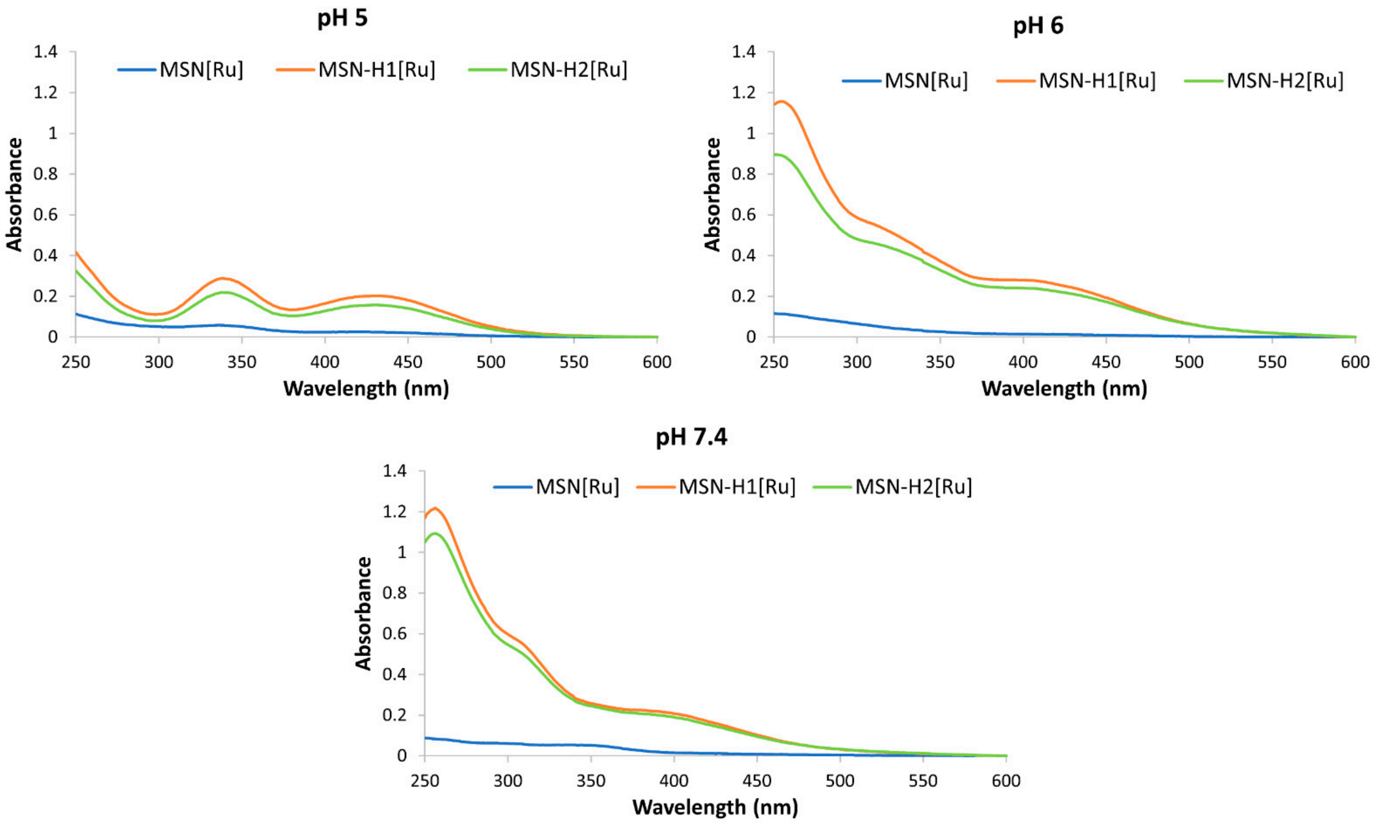

Figure S4. UV/VIS spectra of supernatants at different pH values after 48 h of stirring.

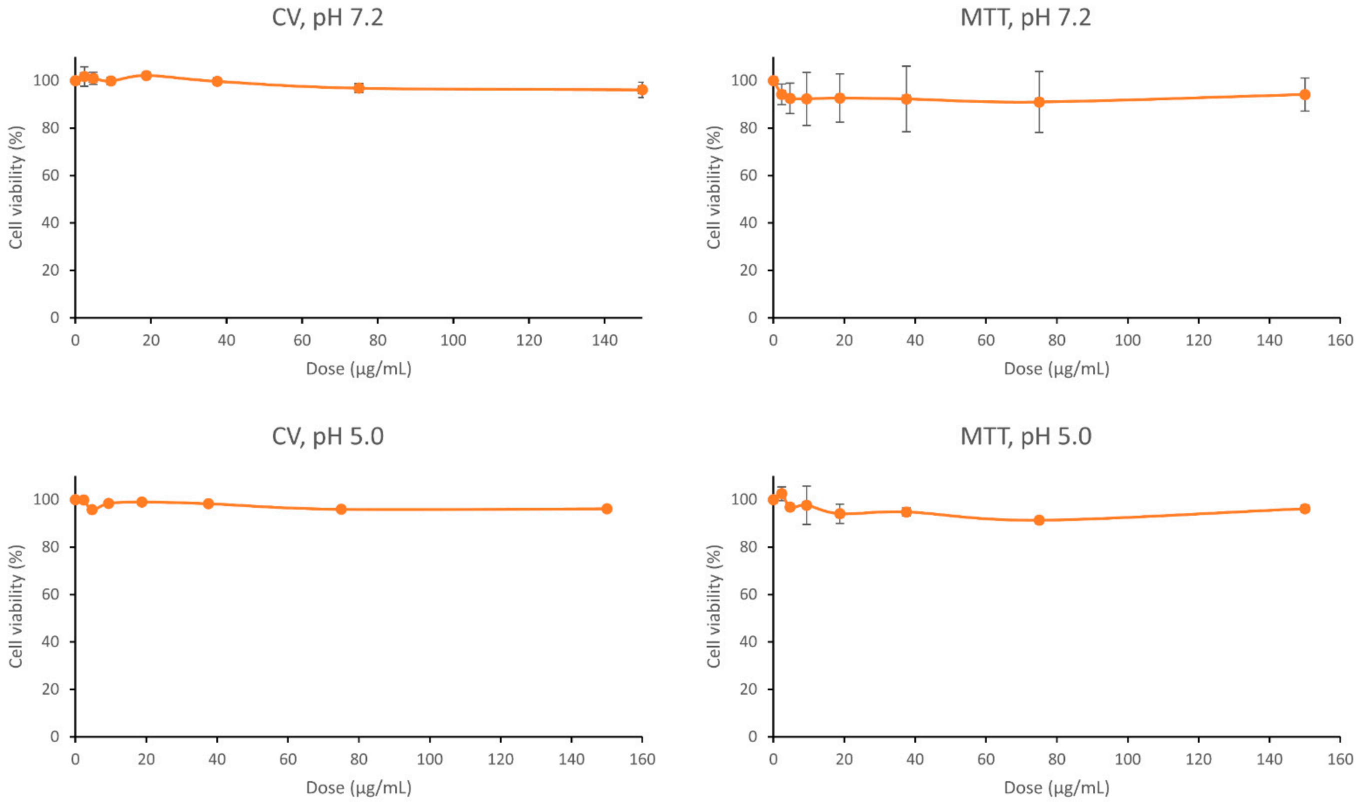

Figure S5. Viability of the B16F1 cells determined with CV and MTT assays treated (48 h) with pristine MSN on pH 5.0 and 7.2.
